# Red-Leafed Lettuces: Genetic Variation or Epigenetic Photomorphogenesis?

**DOI:** 10.3390/plants14030363

**Published:** 2025-01-25

**Authors:** Natalya V. Smirnova, Ivan A. Timofeenko, Konstantin V. Krutovsky

**Affiliations:** 1 Institute of Soil Science and Agrochemistry, Siberian Branch of the Russian Academy of Sciences, Prospect Lavrenteva 8/2, 630090 Novosibirsk, Russia; nat-smirnova@yandex.ru; 2 Interdisciplinary Laboratory of City Farming, Institute of Gastronomy, Siberian Federal University, 660041 Krasnoyarsk, Russia; ivan@timofeenko.com; 3 Department of Forest Genetics and Forest Tree Breeding, Georg-August University of Goettingen, 37077 Goettingen, Germany; 4 Center for Integrated Breeding Research, George-August University of Goettingen, 37075 Goettingen, Germany; 5 Laboratory of Population Genetics, N.I. Vavilov Institute of General Genetics, Russian Academy of Sciences, 119333 Moscow, Russia; 6 Scientific and Methodological Center, G.F. Morozov Voronezh State University of Forestry and Technologies, 394087 Voronezh, Russia

**Keywords:** antioxidants, anthocyanins, environmental factors, epigenetics, flavonoids, gene expression, light, photomorphogenesis, phytochemicals, red-leafed lettuces

## Abstract

Red-leaf lettuces, rich in bioactive compounds like anthocyanins and flavonoids, offer health benefits by reducing oxidative stress and boosting immunity. This article provides an extensive review of the genetic, epigenetic, environmental, and technological factors influencing anthocyanin biosynthesis and leaf coloration in red-leaf lettuce, emphasizing its significance in agriculture and nutrition. The genetics of anthocyanin biosynthesis, environmental influences, practical applications, agronomic insights, and future directions are the main areas covered. Anthocyanin accumulation is regulated by structural, regulatory, and transporter genes, as well as the MYB-bHLH-WD40 (MBW) complex. Mutations in these genes impact coloration and stress responses. Advances in genomic studies, such as GWAS and QTL mapping, have identified key genes and pathways involved in anthocyanin biosynthesis, aiding breeding programs for desirable traits. In addition, light intensity, stress conditions (e.g., drought, temperature), and phytohormones affect anthocyanin levels and photomorphogenesis in general. Controlled environments, like vertical farms, optimize these conditions to enhance pigmentation and phytochemical content. LED lighting and tailored cultivation techniques improve color intensity, antioxidant capacity, and yield in controlled settings. Sustainable production technologies for red-leaf lettuce in vertical farms are being developed to meet consumer demand and promote functional foods, integrating genetic, epigenetic, and environmental research into agronomy. This review highlights red-leaf lettuce’s aesthetic, nutritional, and functional value, advocating for innovative cultivation methods to enhance its market and health potential.

## 1. Introduction

The world’s population increasingly needs food products that not only provide nutritional value, but also contribute to improved health, reduced disease, and increased life expectancy [1]. Research data obtained at the Cancer Institute have shown a correlation between increased vegetable consumption and a reduced risk of chronic diseases such as cancer, cardiovascular disease, and age-related decline in activity in older adults [2,3]. Micronutrient deficiencies are a major global public health problem in many countries, with some authors noting that infants and pregnant women are particularly at risk of not receiving essential nutrients [2,4]. Particular attention should be paid to the nutrition of children, since sufficient amounts of macro- and micronutrients are necessary for their full development. It is believed that these health-promoting compounds are associated with the adequate intake of macronutrients and bioactive compounds present in vegetables. Several studies have found that fruits and vegetables are rich sources of antioxidant nutrients and bioactive compounds, including flavonoids, which are involved in neurodegenerative processes [5]. In addition, it has been established that people who frequently consume fruits or vegetables have a reduced risk of chronic metabolic diseases, such as obesity, cardiovascular disease, and diabetes, and the consumption of fruit and vegetable flavonoids provides health benefits, as supported by studies conducted with several plant flavonoids [6,7,8].

The dietary intake of vitamins E and C for 3 years maintains cognitive abilities and slows down their deterioration. It has been found that levels of vitamin E in food and human blood plasma can reduce the risk of developing Alzheimer’s disease (AD). The dietary intake of vitamin C, carotenoids, and flavonoids also reduces the risk of AD. The dietary intake of flavonoids provides a 50% reduction in the risk of dementia [2,6].

Lettuce is one of the most well-known leafy vegetables in the world, with many uses in both cooking and science. In addition, lettuce is an excellent source of bioactive compounds, such as polyphenols, carotenoids, and chlorophyll, with associated benefits for human health [3]. There are many different varieties of lettuce, with colors ranging from green and yellow to deep red, which is due to the different concentrations of chlorophyll and anthocyanins in the leaves [9,10].

Lettuce (*Lactuca sativa* L., family Asteraceae) is one of the most popular vegetables, consumed fresh or in salad mixtures, and has high production and economic value [11,12]. The crop is unpretentious and can be grown both in open and closed ground. In addition, lettuce can be grown in various traditional ways, both on soil and via soilless methods, such as in containers or on vertical rack systems [13]. Consumer interest in lettuce is growing due to its superior visual quality, low calorie content, low disease incidence, and high content of beneficial phytochemicals. According to a report by the Food and Agriculture Organization of the United Nations [14], the production of lettuce in the world increases annually, while it is noted that the main supplier is China (~56%), and in second and third place are the United States (~12%) and India (~4.3%), respectively. Cultivated lettuces come in different types, can differ in the shape and size of the stalk and stem, and can form different heads: oily or crispy (iceberg or cabbage).

The nutritional or nutraceutical properties of lettuce vary depending on growing conditions and abiotic factors. However, a number of studies show that the variety, i.e., its genetic makeup, also greatly influences the properties of lettuce. For example, romaine and leaf lettuce varieties contain higher amounts of ascorbic acid, vitamin A, carotenoids, and folate, while crispheads contain relatively low amounts of these compounds [13]. Differences in the content of primary and secondary metabolites in red and green lettuce leaves were observed between head and leaf types of lettuce [15]. In addition to growing conditions, genetic variation influences the nutritional and phytochemical properties of lettuce [3].

**Figure 1:**
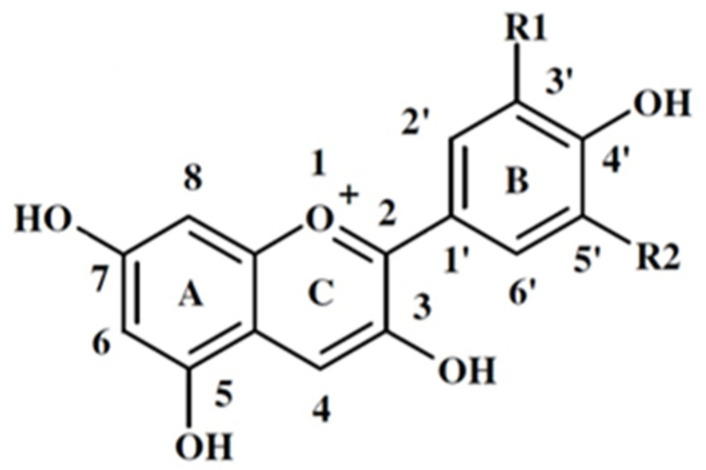
Basic structure of anthocyanidins and anthocyanins with carbon atom numbering (modified from Figure 1 in [16]).

Anthocyanins are a family of naturally occurring flavonoids responsible for variations in the color of leaves, fruits, and flowers of many plants, particularly the red, purple, and bluish colors of lettuce [12]. Red-pigmented lettuce accumulates large amounts of anthocyanins. Anthocyanins are typically present in the form of anthocyanidin glycosides and acylated anthocyanins (Figure 1) [15].

Anthocyanin pigments extracted from plants have been traditionally used as dyes and food colorings to treat various diseases [16]. The most common anthocyanidins in nature are pelargonidin, cyanidin, delphinidin, peonidin, petunidin, and malvidin. The hydroxylation and methylation of the B-ring control the color and stability of anthocyanins. Table 1 lists most of the anthocyanidins and other coloring substances that contribute to the increase in red color under the influence of abiotic factors.

Blueness increases with the increase in the number of hydroxyl groups, while redness increases with the increase in methylation in the B-ring [17,18].

**Table 1:** Anthocyanidins, which are precursors of all anthocyanin compounds.

Anthocyanidin	Side Radicals of the B-Ring		Color	Present in Red-Leaf Lettuce
	R_1_	R_2_		
Anthocyanidins				
Cyanidin (Cy)	HE	N	Orange-red	Cyanidin 3-O-(6′-O-malonylglucoside)—97% of total anthocyanin content [19] or 251–928 mg/kg wet weight [20]
Peonidin (Pn)	OSN_3_	N	Red	
Pelargonidine (Pg)	N	N	Orange	
Malvidin (Mv)	OSN_3_	OSN_3_	Purple	
Delphinidin (Dp)	HE	HE	Blue-red	
Petunidin (Pt)	OSN_3_	HE	Purple	
Carajurin	H	H	-	
Arrabidin	H	H	-	
5-methylcyanidin	HE	OCH_3_	Orange-red	
Peonidin	HE	HE	Orange	
Capensinidin	OH	OCH_3_	Blue-red	
Eupinidin	OH	OCH_3_	Blue-red	
Pulchellidin	OH	OCH_3_	Blue-red	
6-hydroxydelphinidin	OH	OH	Blue-red	
Aurantinidin	OH	OH	Orange	
30-hydroxyarrabidine	H	H	Blue-red	
Tricetinidine	H	OH	Red	
6-hydroxycyanidin	OH	OH	Red	
Rosinidin	Red	Red	Red	
Other substances that color plants				
Quercetin				3-0-(6′-O-malonyl)glycoside [13]
Tartaric acid				2,3–di-O-caffeoyltartaric acid [13]

There are many studies confirming the fact that anthocyanins participate in the prevention of cardiovascular diseases, and a connection has been shown between the level of anthocyanins in lettuce and the manifestation of antioxidant, antidiabetic, anticancer, anti-inflammatory, antimicrobial, antitumor, and antimutagenic effects [1,21,22,23]. However, quantitative data on the main health-promoting metabolites of red-pigmented lettuce are clearly lacking.

It was found in early studies that red lettuce had higher total anthocyanin and phenolic content and antioxidant capacity than green lettuce [24,25,26]. The main difference between red and green lettuce was the anthocyanin content. Mulabagal et al. [27] compared the in vitro biological activity of green and red lettuce and showed that the aqueous extract of red lettuce has a higher biological activity and contains more anthocyanins compared to green varieties. In addition, the presence of only one major anthocyanin was shown by HPLC analysis of the red lettuce extract, and it was characterized as cyanidin-3-O-6-malonyl-β-glucopyranoside. Subsequent glycosylation results in a red shift in the color of the anthocyanin with increased stability. While 3-glycosides increase stability, 5-glycosides tend to decrease stability. Moreover, the attachment of 5-glycosides to anthocyanidin can also lead to the formation of colorless pseudobases since the loss of the hydroxyl group at position 5 makes the anthocyanin more susceptible to hydration reactions. Sugar residues in anthocyanins are often acylated with aromatic or aliphatic acids. Common aliphatic and aromatic acids involved in acylation reactions result in color changes (blue shift) and increased stability due to intra- and intermolecular copigmentation reactions. In addition to the biosynthetic genes involved in anthocyanin pigment formation, vacuolar pH and cell shape have a negative effect on anthocyanin pigments. For example, in petunia flowers, the acidification of the vacuole causes a red color change, while mutations affecting pH result in a blue color change. Even with high pigment accumulation, plants may appear colorless due to the shape of their cells. This is due to the differences in reflected light between conical and flat cells [28].

It was found that the variety, agricultural practices, and growing conditions can change the content of phytocomponents, leaf color, and the quality of lettuce [29,30]. The diversity of physiological characteristics of lettuce tissues, pigment forms, and the characteristics of their distribution in cells contribute to the fact that phytochemicals and the antioxidant capacity of a given crop can differ even in the outer and inner leaves of plants. Thus, it was found in [31] that total soluble solids (TSSs) and color values of green and red lettuce cultivars were significantly dependent on leaf position on the plant: internal leaves showed significantly higher levels of TSSs than average, and the outer leaves of lettuce were both red and green. The average TSS content in green varieties was significantly higher than that of red varieties. The color of the leaves can also vary depending on their position for each lettuce variety, which occurs as a result of the breakdown of chlorophyll and the accumulation of anthocyanins: the values indicating the lightness of the color gradually decreased from the inner to the outer leaf. Similar patterns were recorded for the indices indicating the color when the leaf color changed from green to red: the outer leaves of green varieties showed lower (more negative) values due to high chlorophyll content, with a darker green color, while the outer leaves of red varieties showed higher values of this indicator due to the increased content of anthocyanins, with a red color.

The influence of leaf position on antioxidant activity was demonstrated for the first time in [32]. The nutrient content of several anthocyanin-rich red and green lettuce varieties was compared and quantified: green and red lettuce varieties showed different characteristics, mainly due to the presence of anthocyanins in red varieties. It was shown that in red lettuce leaves, the outer leaves had the highest content of phytonutrients and antioxidant properties. The results of these studies have practical and scientific value, including the use of the identified patterns in drawing up breeding programs aimed at creating new varieties and developing functional human nutrition systems, as well as for the pharmaceutical industry.

**Figure 2:**
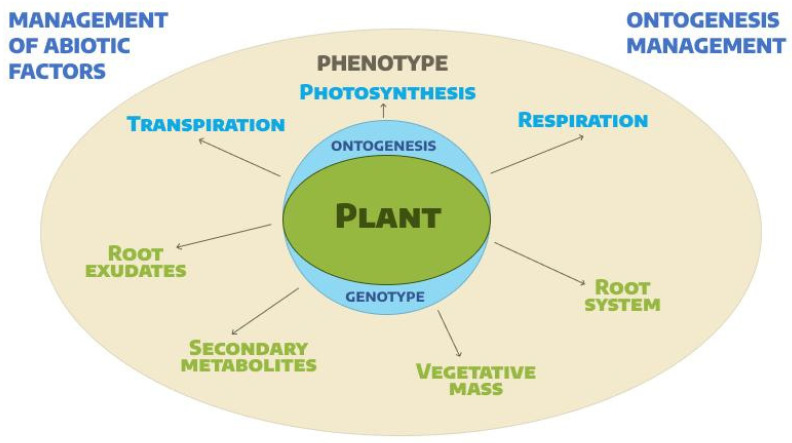
The diagram demonstrates the relationship between environmental conditions and the individual characteristics of a plant at all stages of its development: the individual development of each crop of a certain variety or species is determined by the genetic characteristics of the individual, which are manifested in the activity of the main biological processes: respiration, photosynthesis, transpiration, metabolic processes, and the accumulation of nutrients in tissues and different parts of the plant. In addition, all processes are also under the influence of abiotic and biotic factors that determine the speed and rate of the growth and development of plants, changes in phenophases, the rate of seed maturation, and the quality of plant raw materials for various purposes.

Thus, the food significance and nutritional value of red-leafed plants have been identified and studied quite well. The results of a number of studies [11,18,33,34,35] are schematically generalized in Figure 2, which shows that the individual development of each separate species or variety of lettuce is determined by the genetic characteristics of the individual, which are manifested in the activity of the main biological processes of plants: respiration, photosynthesis, transpiration, metabolic processes, and the accumulation of nutrients in tissues and different parts of the plant. In addition, all processes are also under the influence of abiotic and biotic factors that determine their growth and development, changes in phenophases, the rate of seed maturation, and the quality of plant products for various purposes.

In the following sections, this review also focuses on the different factors—genetic, epigenetic, or environmental—that affect the photomorphogenesis of red-leafed lettuce. Photomorphogenesis is the process by which plants regulate their growth, development, and morphology in response to light signals. It involves changes in plant structure and function triggered by the perception of light, enabling plants to adapt to their environment and optimize photosynthesis. Key features of plant photomorphogenesis include (1) light sensing via specialized photoreceptors to detect various wavelengths of light, such as phytochromes (detect red (R) and far-red (FR) light), cryptochromes (detect blue light), phototropins (mediate responses to blue light and UV-A), and the UV resistance locus 8 (UVR8) photoreceptor (senses UV-B light); (2) light-dependent developmental changes, such as de-etiolation (transition from dark-grown (etiolated) to light-grown morphology, including, for instance, the shortening of hypocotyls, expansion of cotyledons, and initiation of chlorophyll synthesis) and shade avoidance response (adaptations to reduced red-to-far-red light ratios, such as the elongation of stems to outgrow shading by neighboring plants); (3) the epigenetic regulation of genes linked to light-responsive pathways and processes, such as, for example, the activation of genes related to photosynthesis and the suppression of growth-related genes in darkness; and (4) coordination with other hormonal pathways and plant hormones like auxins, gibberellins, and abscisic acid that modulate growth responses under varying light conditions. Photomorphogenesis ensures optimal light capture for photosynthesis, allows plants to compete for light in crowded environments, and regulates transitions between life stages, such as germination, flowering, and seed production. By integrating light cues, photomorphogenesis is crucial for plant survival and productivity in diverse environmental conditions.

## 2. The Influence of Light on Leaf Color and the Antioxidant Activity of Lettuce

It is known that light controls photosynthesis and regulates the morphology, physiology, and phytochemical composition of plants. Plants use light as an energy source for photosynthesis, signaling, and many other fundamental processes; therefore, primary and secondary metabolism are regulated by the quantity and quality of light entering the leaf blade [12]. Compelling evidence has been provided that anthocyanin production in plants can be increased by altering the quality and quantity of light during plant growth [36,37].

The manipulation of light for plant growth is one of the effective ways to regulate plant growth in growth chambers and greenhouses, where different artificial lighting conditions are often used. For this purpose, Ali et al. [38] conducted experiments where two varieties of lettuce, green and red, were treated with two different LED treatments: (1) continuous LED, in which the average photosynthetic photon flux density (PPFD) at seedling level was maintained at 228 μmol s^−1^ m^−2^, with a photoperiod of 16 h for a growth cycle of 30 days, and (2) pulsed LED treatment in a dynamic mode with the pulse frequency set at 1 kHz, with a duty cycle of 50% along with PPFD at 228 μmol s^−1^ m^−2^ and a 16 h photoperiod. A significant decrease in the average fresh weight of both varieties was observed under pulsed LED lighting, while a significant increase in leaf length was noted among the red lettuce treatments. Both treatments resulted in minor changes in photosynthetic pigments—total chlorophyll and carotenoids—while no significant differences were observed in the phenolic index values or anthocyanin production of green lettuce. However, red lettuce showed a significantly higher phenolic index for continuous LED lighting.

According to modern concepts, plants have at least five groups of photoreceptors that perceive information not only about lighting conditions and daylight hours, but also about ambient temperature, the presence of pathogens or competing neighbors, the direction of the gravity vector, and other factors [39,40]. These receptors include red (RL) and far-red (FRL) receptors (phytochromes); receptors that perceive ultraviolet A radiation, as well as blue (BL) and green (GG) light (cryptochromes, phototropins, and proteins of the ZEITLUPE family); as well as a receptor for ultraviolet B radiation (UVB) (the UVR8 protein) [41].

**Figure 3:**
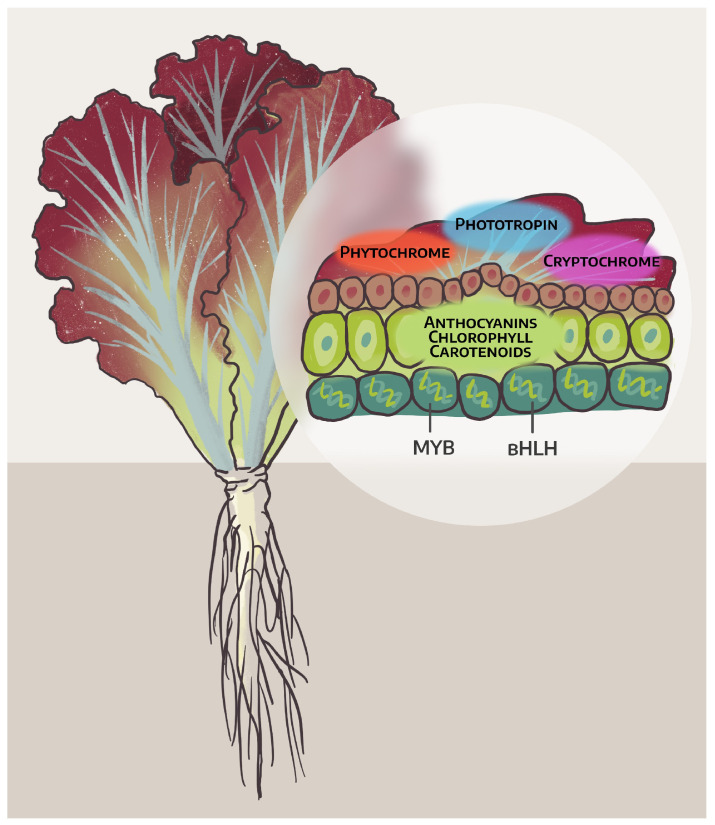
Light-sensitive system of red-leaf lettuce, providing different productivity and product quality.

Using light-emitting diodes (LEDs), it is possible to create individual spectra, including a spectrum simulating sunlight, which allows for significant changes in the course of ontogenesis, as well as the growth and development of crops. Considering the fact that the spectrum of light can influence the physiological reactions of plants, it is already possible today to influence different stages of plant growth by means of several “fixed” spectra, which makes dynamic spectral manipulation possible, including changes in the mechanisms of functioning of light recipes throughout the growth cycle of lettuce (Figure 3).

As has been established previously and confirmed by modern methods, plants use photosynthetic pigments in their leaves to capture photosynthetic active radiation (PAR) to initiate the synthesis of sugar molecules. These photosynthetic pigments are present around the thylakoid membranes of chloroplasts and serve as the primary electron donors in the electron transport chain [42]. Specifically, photosynthetic pigments absorb light and transfer energy via resonance energy transfer to a specific chlorophyll pair in the reaction center of photosystem II P680 or photosystem I P700. In this light-dependent reaction, water molecules are broken down to generate ATP and NADPH and release oxygen molecules as a byproduct. It has been established that in a subsequent light-independent reaction in the chloroplast stroma, the energy from ATP and electrons from NADPH are used to convert carbon dioxide into glucose and other products via the Calvin cycle.

In [29,43], the results of studies assessing the growth, development, and phytochemical composition of red and green crops grown hydroponically under different exposition spectra are presented. One of spectra simulated the sun (SUN), and six others were conventional light spectra used in indoor growing systems: 5% ultraviolet-A (UV-A), 20% blue (B), 26% green (G), 26% red (R), and 23% far-red (FR) light, as percentages of photon flux density (PFD). Fluorescent white light (FL—6500 K) was used as a control. Plants received 200 ± 0.7 μmol m^−2^ s^−1^ of biologically active radiation (300–800 nm) for 18 h and were grown at a temperature of 20.0 ± 0.2 °C. The results indicate that the dry mass of plants under SUN treatment was not significantly different from that under red-blue light treatment at all harvest times; considering the dry mass of plants at day 17, the leaf area of lettuce grown under B + R conditions was 15–39% larger than that of those grown under blue and fluorescent white. The leaf area at day 42 was 39–78% larger in the 100B, SUN, and FL treatments than in the B + R treatment. The increased influence of LED-simulated solar light resulted in the bolting and flowering of lettuce in the SUN treatment. In addition, the authors noted that both total phenolic and anthocyanin concentrations in lettuce leaves were higher in the B + R treatments than in the SUN, 100R, and 100B treatments. These studies further confirm the basic information on lettuce responses to the LED-simulated solar spectrum compared to conventional B + R treatments and provide insight into lettuce growth changes and morphology under different spectra.

As the authors note, amid the flood of studies on the effects of light spectra on lettuce growth, photoperiod is a relatively unexplored parameter in studies determining the effect of lighting on indoor leafy greens. This is likely due to the less pronounced effect of photoperiod on growth and nutrient accumulation in leaves compared to the quality and quantity of light received by the plant. It is noted that photoperiods of 14–18 h of illumination are usually used when growing green crops, which is already an optimal level. Thus, any deviation from this established norm does not bring significant benefits during the vegetative growth of leafy greens. Despite the fact that all tested daylight integrals are only a fraction of full sunlight, leafy greens grow well in these so-called low-light conditions. Yudina et al. [44] developed a model to predict plant productivity to answer the following question: Does increasing light duration stimulate plant productivity without causing changes in photosynthesis and respiration? The results of their study showed that increasing light duration can stimulate dry weight accumulation and that this effect can also be caused by increasing the photoperiod with decreasing light intensity. Therefore, the authors showed that increasing light duration is an effective approach to stimulating lettuce production under artificial lighting.

As shown by the above studies, higher illumination can lead to higher biomass, but phytonutrient accumulation can be active at lower illumination. Furthermore, electric lighting can represent a significant portion of production costs, and therefore there is little incentive to use high PPFD or a long photoperiod in a closed production agroecosystem to make such a system economically viable and environmentally sustainable [12,45].

Rong Sng et al. [46] found that the morpho-anatomical traits of *Lactuca sativa* cv. Batavia plants grown for three weeks in a BL environment had a higher leaf area and shorter petioles compared to lettuce grown in polychromatic or monochromatic lights, such as RL and FR light.

Every year, more and more research is being conducted in the field of photobiology and photomorphogenesis, but the question of what mechanisms can be used to obtain a redder or greener lettuce while simultaneously increasing antioxidant capacity and nutritional value for the consumer has not been answered. A number of authors (e.g., [47,48]) noted that plant growth, including in lettuce, occurs as a result of biomass accumulation following the trajectory of a sigmoid curve. The choice of the plant growth stage for experiments with the light spectrum can significantly affect the reaction, and vice versa, the light spectrum can be used to create the desired morphological characteristics. Lettuce seedlings should be compact plants with a good root system and a large leaf mass area. After rooting the seedlings, when treated with light emitters, rapid leaf growth occurs to increase light capture [18].

Low light intensity affects the dormancy of the anthocyanin pathway in red-leaf lettuces, and conversely, high light intensity enhances the expression of genes associated with anthocyanin biosynthesis, including regulatory genes (*PAP1* and *PAP2*) and structural genes (*CHS*, *F3H*, *DFR*, and *LDOX*). The accumulation of anthocyanins was negatively affected by switching from fluorescent to LED lighting: the molecules decreased under white and red-blue LED illumination. Red light and UV-C radiation can inhibit the accumulation of anthocyanins or cause their degradation. At the same time, studies have shown that both blue and red light effectively stimulate the formation of anthocyanins in strawberry fruits [49,50].

Thus, lettuce responds positively to changes in the light spectrum and photoperiod, and it is noted that due to these parameters, it is possible to change not only the aboveground biomass and morphometry, but also agronomically important traits, such as dry matter, the content of photosynthetic pigments (primarily anthocyanins), and antioxidant activity. In lettuce grown at an increased level of irradiation in the range from 250 to 300 μmol/m^2^, an increase in the concentration of anthocyanins was obtained [47,51]. The effect of spectrum on growth and taste depends on the lettuce variety. By using only red light, it is possible to control the absorption of nutrients, and therefore the quality and taste of the grown produce. Using wavelengths of light emitters in the range from 620 nm to 700 nm has several positive effects on the nutritional value of lettuce: the concentration of ascorbic acid decreases, antioxidant properties increase, and the absorption of N, K, Ca, and Mg by the plant root system is stimulated, which immediately affects the accumulation of sugars and other components. Both far-red and blue LEDs have promising prospects for use as supplemental lighting and for controlling quality and growth.

Rossi Pinheiro et al. [52] observed that silver-colored photo-selective nets or coverings, reflecting a large amount of incident solar radiation, strongly altered the microclimate under the net, thereby leading to changes in leaf transpiration as well as photosynthetic pigments in lettuce. In addition, it was noted that supplemental BL (250 μmol s^−1^ m^−2^) increased flavonoid content and phenolic accumulation in several species, including red-leafed lettuce [53].

However, the influence of lettuce varietal characteristics on the efficiency of anthocyanin and other nutrient synthesis under modified artificial lighting conditions remains poorly understood [18], which means that studying the effect of photon flux density on the accumulation of anthocyanins is very promising and useful.

## 3. Effect of Temperature on Leaf Color and the Antioxidant Activity of Lettuce

Plant growth and development are influenced by various epigenetic or environmental factors, including light, temperature, CO_2_, water availability, and pathogens. External environmental factors often trigger abiotic and biotic stress responses in plants, including the production of secondary metabolites, which play a key role in stress tolerance. However, the additional production of secondary metabolites requires energy expenditure for synthetic processes, and these costs can lead to a decrease in plant growth and development [13]. Mutant plants responding to constitutive biotic stress showed a smaller size and weight of aboveground biomass due to the activation of adaptation reactions and resistance development through the synthesis of salicylic acid, including the production of antimicrobial secondary metabolites [54]. This process is very important in the development of mechanisms for controlling plant growth processes and color. Accordingly, to increase the production of metabolites by a plant through responses to the environment, it is necessary to take into account most of the abiotic factors that affect plant growth and development, one of which is the ambient temperature of both roots and aboveground biomass. It was found that the optimal temperature for anthocyanin biosynthesis is in the range of 15–30 °C. However, plants can actively synthesize anthocyanins at lower and higher temperatures. For example, under low-temperature stress (4 °C), the regulation of the expression of *FaMYB10* and *FaMYB1* genes was noted, which significantly increased the activity of structural genes and led to an increased accumulation of anthocyanins in strawberry leaves [18,55].

Analyzing the data obtained by a number of scientists, it is possible to identify another mechanism for regulating the growth and amount of plant pigments, as well as minimizing crop losses—by changing the temperature of the root zone of plants (RZT) at different stages of their growth. This suggests that RZT control may be a relevant method, especially in vertical farms and greenhouse complexes where flood tables or a flooding system are used. For instance, Levine et al. [56] showed that the exposure of red-lettuce roots to low temperature significantly reduced leaf area, stem diameter, and above- and below-ground fresh weight. The results suggest that low-temperature root treatment triggers stress responses throughout the plant, resulting in reduced leaf and root growth.

However, in another study, heating the root zone by heating the solution in the root zone did not result in significant changes in plant biomass [57]. Cooling the root zone to 20 °C increased the biomass of aeroponic lettuce compared to plants under ambient conditions (24–38 °C) in a tropical greenhouse [58].

The production of various plant metabolites is influenced by root zone temperature in many plants. Studies conducted in [56,59] showed that human-preferred compounds, such as anthocyanin, phenolics, and sugars, increased significantly in red lettuce leaves when their roots were exposed to low temperature. Sugar accumulation was observed in spinach, cotton, and tomato when roots were exposed to low temperature. In contrast, the exposure of roots to high temperature did not change the anthocyanin, phenolics, or sugars in leaves. However, increasing soil temperature using electric heating cables suppressed anthocyanin and sugar accumulation in lettuce leaves under field experimental conditions.

Root stresses such as drought or salinity cause plant growth limitations, followed by a reduction in leaf photosynthetic capacity (see Box 1 in [60]). Reduced plant growth and photosynthesis were observed when plants were exposed to low root temperatures [61,62]. Low root temperature treatment resulted in decreased water uptake by lettuce, which resulted in a reduction in photosynthesis in the shoot growth zones. However, sugar content in both lettuce leaves and roots increased following low root temperature treatment, while nitrate concentrations, for example, changed significantly only in leaves where the temperature was reduced to 40% of that in plants under ambient conditions. Considering that photosynthetic nitrate uptake is likely not increased in leaves due to low root temperature stress, the suppression of nitrate transport from roots to leaves may be responsible for the decrease in nitrate concentration in the leaves themselves [57].

RZT can also significantly affect the plant metabolite contents in both leaves and roots [62]. The concentrations of 19 amino acids (isoleucine, serine, tyrosine, valine, methionine, leucine, cysteine, phenylalanine, threonine, homoserine, histidine, pyroglutamate, alanine, glutamate, glutamine, ornithine, glycine, β-alanine, and asparagine) were significantly increased in lettuce roots under 15 °C treatment compared to lettuce with its root system maintained at a solution temperature of 25 °C. At the same time, the morphometry of the leaves was not significantly changed. In contrast, compared with the 25 °C treatment, 35 °C treatment significantly affected the metabolite contents in both the roots and leaves. The roots showed significant increases in 7 sugars (glucose, fructose, sucrose, turanose, lactose, ribose, and maltose), 21 amino acids (arginine, phenylalanine, lysine, glycine, histidine, proline, cysteine, isoleucine, threonine, tyrosine, glutamine, valine, asparagine, pyroglutamate, β-alanine, 3-cyanoalanine, leucine, tryptophan, ornithine, serine, and methionine), and 5 metabolites in the tricarboxylic acid cycle (TCA) (isocitric acid, 2-xoglutaric acid, citric acid, fumaric acid, and malic acid).

In *Arabidopsis*, the overexpression of a plasma membrane water channel protein mitigated the low-root-temperature-induced decline in hydraulic activity and plant growth, suggesting a role for water uptake in root temperature stress. Since drought stress triggers various secondary metabolic pathways, the accumulation of anthocyanins, phenolics, and sugar in leaves in the experiment may have been a response to drought stress: a study in green crops showed that drought stress applied to roots can lead to the accumulation of phenolics and sugar in hydroponically grown lettuce seedlings [63,64].

It was noted in [34,56] that photosynthesis impairment caused by abiotic stress often accompanies oxidative stress. Plants exposed to drought, salinity, and low temperature experience oxidative stress, which is followed by a decrease in photosynthetic capacity and oxygen emission, which is associated with the functioning of cofactors in photosystem II (PSII), and as a consequence, this leads to limitations in plant biomass production. Salinity stress induces oxidative stress in leaves, resulting in the production of reactive oxygen species (ROS), the activation of antioxidant enzymes, and leaf chlorosis. As shown in [65], the low-temperature treatment of lettuce roots causes the accumulation of hydrogen peroxide in leaves, accompanied by lipid peroxidation. This finding indicates that low-temperature stress in the root zone induces oxidative stress in leaves, presumably by limiting photosynthesis. Plants cope with oxidative stress by producing antioxidant metabolites, including phenolic compounds such as anthocyanin. As a result of stress, anthocyanin content and phenolic content in leaves increase when roots are exposed to low temperature, indicating an antioxidant role of these metabolites in response to elevated hydrogen peroxide levels in leaves.

It was found that lettuce exposed to low air temperature at a level of 10–15 degrees showed an increased production of anthocyanins and polyphenols—which led to a change in the color intensity of the lettuce leaves—and also an increased ability to remove DPPH radicals. The *Arabidopsis* MYB transcription factor AtMYB60 functions as a transcriptional repressor of anthocyanin biosynthesis genes in lettuce [34]. Given that MYB family proteins are common regulators of the anthocyanin synthesis pathway in many plants [66], anthocyanin accumulation in lettuce leaves induced by low root-zone temperature is also regulated by intrinsic MYB transcription factors. To enhance the levels of secondary metabolites, the regulation of these transcription factors may be important for the production of crops with improved product quality.

It was shown in [67,68] that treatment with a solution with a high RZT contributed to the improvement of pigment content, but negatively affected plant growth. In this experiment, plants were grown in five temperature variants of RZT: 25 °C; HT1 (12 days at 25 °C, then 4 days at 35 °C); HT2 (8 days at 25 °C, then 8 days at 35 °C); HT3 (4 days at 25 °C, then 8 days at 35 °C); and 35 °C at an air temperature of 22 °C. It was observed that an increase in the duration of RZT treatment at 35 °C led to a decrease in the dry weight of the shoots and roots, and an increase in the content of chlorophyll, carotenoids, and anthocyanins. In addition, the chlorophyll content increased significantly when treated with RZT at 35 °C for more than 4 days, while the carotenoid and anthocyanin contents increased significantly when treated with RZT at 35 °C for more than 8 days. It was also found that RZT treatment at 35 °C compared with 25 °C limited plant growth in the roots and leaves due to decreased root growth, but increased pigment content, including anthocyanin, chlorophyll, and carotenoids. This was probably due to the typical response to heat stress. The longer the period of exposure of the root zone to high temperature (35 °C), the more the anthocyanin, carotenoid, and chlorophyll contents increased, while the shoot dry weight decreased, indicating gradual plant mortality before harvest. However, increasing the pigment levels may be an effective mechanism for controlling leaf color in lettuce. Plants typically accumulate ROS under stressful conditions, and plant stress tolerance is often associated with an increased activity of antioxidant enzymes. The increase in anthocyanins and carotenoids in leaves at RZT 35 °C is due to the formation of ROS in leaves as a response to high-temperature stress and an increase in antioxidants to remove them.

In addition, the authors noted in [67,68] that carotenoids are produced from β-alanine via acetyl- CoA, but the leaves of the plants in the 35 °C experiment were enriched in β-alanine, and it is possible that β-alanine metabolism influenced carotenoid accumulation. The root zone temperature significantly affected the concentrations of elements in both the leaves and roots. Compared with the 25 °C treatment, plants in the 15 °C treatment experienced a significant decrease in Ca, Mn, As, and Cd in the roots, with a significant increase in P, K, Li, and Rb and a significant decrease in Cu and Cs in the leaves. Compared with the 25 °C treatment, the 35 °C treatment resulted in a significant increase in Fe, Zn, Mo, Cu, Ni, Co, Li, Se, and Cd and a significant decrease in Ca, S, Mn, As, and Sr in the roots. Simultaneously, with the 35 °C treatment, there was a significant decrease in P, K, Ca, Mg, S, Fe, Mn, Zn, Mo, Cu, Na, Ge, As, Se, Rb, Sr, and Cs in the leaves.

Gazula et al. [69] studied three closely related red-leafed Lollo Rosso cultivars [‘Lotto’, ‘Valeria’ and ‘Impuls’ (Nunhems Seed, Haelen, The Netherlands)], which differed mainly in the number of genes controlling anthocyanin levels. ‘Lotto’ (one gene for color intensity) is the best breeder of ‘Lollo Bionda’ and ‘Lollo Rosso’, ‘Valeria’ (two genes) is a redder mutant of ‘Lotto’, and ‘Impuls’ (three genes) is a redder mutant of ‘Valeria’. Plants were grown in chambers with temperature regimes of 20 °C day/night (D/N), 30/20 °C D/N, and 30 °C D/N. Regardless of the cultivar, anthocyanin and chlorophyll b concentrations were highest, moderate, and lowest at 20 °C D/N, 30/20 °C D/N, and 30 °C D/N, respectively. Anthocyanin and chlorophyll b concentrations were also found to decrease in the order of ‘Impuls’ (three genes) > ‘Valeria’ (two genes) > ‘Lotto’ (one gene), regardless of temperature. These data provide evidence that pigment concentrations in lettuce leaves are inversely related to ambient temperature during plant growth. The data also suggest that low temperatures during the dark phase may mitigate the heat-induced decrease in lettuce leaf pigment levels [69].

In summary, low and high temperatures in the root zone limit plant growth through various internal mechanisms, and implementing high temperatures in the root zone four days before harvesting can slightly reduce the production process, but lead to the accumulation of pigments, which in turn contributes to an increase in the added value of lettuce and color. However, in general, this direction and its practical application remain poorly understood and should be studied further.

## 4. Effect of pH on Leaf Color and the Antioxidant Activity of Lettuce

Anthocyanins are potent antioxidants in plants and maintain an optimal cellular redox equilibrium by neutralizing free radicals with their hydroxyl groups to reduce oxidative damage [70]. Anthocyanins are derived from anthocyanidins, which are structurally based on the flavylium ion (2-phenylchromenylium). This flavylium ion is responsible for the red color of anthocyanins at low pH. At neutral pH, the flavylium cation undergoes deprotonation to form a resonance-stabilized quinoid base, which appears purple. With further increases in pH, the quinoid base can form anionic species that appear blue. The predicted values of the acid dissociation constant (pK_a_) for these transitions are about 1–3 for the flavylium cation, 4–5 for the quinoid base, and 7.5–8 for the quinoid monoanion [16]. In addition, the specific color of the anthocyanin also depends on the substituents in the B-ring, with hydroxyl groups tending to shift the color toward blue, and methoxyl groups toward red. For example, delphinidin (with three hydroxyl groups) appears blue, while pelargonidin (with one hydroxyl group) appears red [17].

**Figure 4:**
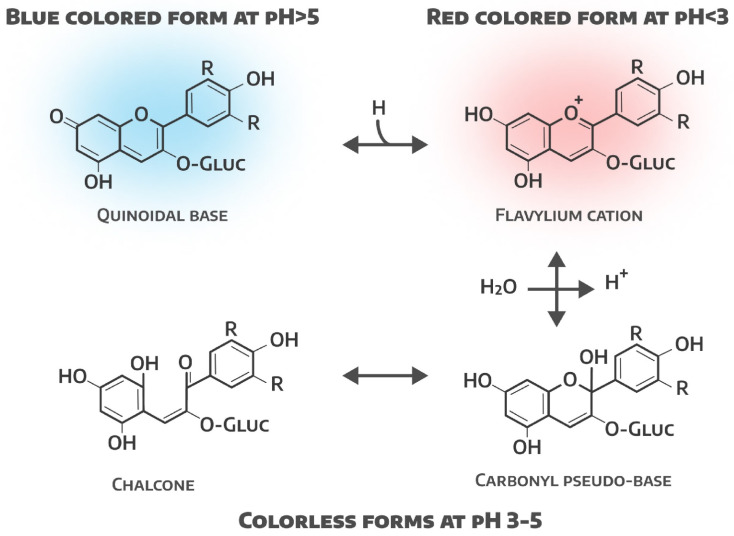
Conversion of anthocyanins into interchangeable forms at different pH levels (adapted from Figure 4 in [71]).

Anthocyanins are water-soluble. However, they exhibit a very interesting chemistry in aqueous solutions, and can present four main interconvertible species with different relative amounts at a given pH (Figure 4).

At low pH, the flavylium cation is most prominent and has a deep red color. As the pH increases, the anthocyanins are converted to colorless forms such as pseudobases and chalcones, and at pH > 5, the anthocyanin changes to a blue quinoid form. These anthocyanidins are further attached to sugars such as glucose, galactose, and rhamnose via an α/β linkage exclusively at position 3 of the aglycone. Alternatively, they can also be acylated with cinnamic acid, caffeic acid, ferulic acid, malic acid, oxalic acid, and succinic acid, to name a few [72].

It has been established that, in an acidic environment, the color of red-leafed lettuces changes due to the internal rearrangement of pigments, but in parallel with this, an oppositely directed process occurs, in which the antioxidant activity of the lettuce extract increases with increasing pH, which can be used in practice when growing red-leafed lettuces with given parameters of color and quality.

## 5. Bioactive Phytochemicals and Metabolites of Lettuce

Lettuce is an important dietary source of bioactive phytochemicals. The screening and identification of health-promoting metabolites, as well as assessing relationships with phenotypic traits, may help consumers adjust their preferences for lettuce plant types. Modern metabolomics analysis methods allow the investigation of key health-promoting individual metabolites and the antioxidant potential of agricultural plants. Assefa et al. [73], using UPLC-DAD-QTOF/MS (TQ/MS) and UV–visible spectrometry instruments, identified and quantified three anthocyanins, four hydroxycinnamoyl derivatives, two flavonols, and one flavone in 113 samples of germplasm and commercial cultivars of lettuce at the mature stage. Total phenolic content (TPC) and 2,2-azino-bis-(3-ethylbenzothiazoline-6-sulfonic acid) (ABTS) radical absorbance potentials were estimated, and the relationships between the biochemical and phenotypic traits of 113 lettuces were investigated. The metabolite contents varied significantly among the lettuce samples: cyanidin 3-O-(6-O-malonyl) glucoside (4.7–5013.6 μg/g DW), 2,3-di-O-caffeoyltartaric acid (337.1–19,957.2 μg/g DW), and quercetin 3-O-(6-O-malonyl) glucoside (45.4–31,121.0 μg/g DW) were the most dominant in the red-pigmented lettuce samples, and hydroxycinnamoyl and flavonol derivatives were the most abundant anthocyanins. Lettuces with dark to very dark red-pigmented leaves, round leaf shape, strong leaf waviness, and very tight leaf cuts were found to have high levels of flavonoids and hydroxycinnamoyl derivatives. Key variables were identified that could make a major contribution to plant breeders developing varieties with improved bioactive compounds and to nutraceutical companies developing nutrient-dense foods and pharmaceutical formulations.

Assefa et al. [73] systematized the traits and quality of lettuces grown in the field and laboratory. Morphological traits were assessed based on the modified International Union for the Protection of New Varieties of Plants (UPOV) descriptors for lettuce: leaf length, width, and lettuce plant weight. Other qualitative morphological traits, such as cotyledon color, plant growth type, leaf shape, leaf position, leaf blade (degree of edge waviness and density of marginal notches on the apical part), and intensity of red coloration on the outer leaves, were also investigated. About 76% of the samples contained red color at the cotyledon stage, while the remaining lettuce samples did not contain red color. Another important characteristic was the intensity of the red coloration of the outer leaves. The intensity of red coloration was assessed on the outer leaf and ranked from 1 to 5 (very light, light, medium, dark, and very dark, respectively). Most of the samples had medium and light intensity, accounting for 37.2% and 31.9% of the total resources. The remaining 16.8%, 10.6%, and 3.5% of the samples had dark, very light, and very dark red color intensity, respectively. Total phenolic content (TPC) and antioxidant potential were quantified using spectrophotometry. As noted by the authors of the study, significant variations in the radical reduction potentials of TPC and ABTS were observed among genetic varieties [73].

Llorach et al. [74] reported TPC contents ranging from 18.2 to 571.2 mg/100 g FW and ABTS antioxidant potential ranging from 61.3 to 647.8 mg TEAC/100 g FW for five lettuce and escarole “frisser” cultivars. In another study, TPC contents assessed in five lettuce cultivars ranged from 13,900 to 46,900 μg GAE/g DW; red-leaf cultivars showed the highest amounts. Differences were found between green and red lettuce and escarole: caffeic acid derivatives were the main polyphenols in green varieties, while flavonols were observed in higher amounts in red varieties and escarole, and anthocyanins were present only in red-leafed varieties. Moreover, lettuce and escarole showed differences in flavonol composition, as quercetin derivatives were observed only in lettuce samples, while kaempferol derivatives were found only in escarole samples. In general, red-leafed vegetables showed higher levels of both flavonol and caffeic acid derivatives than green lettuce and escarole varieties. Regarding vitamin C, red-leafed lettuces showed higher contents than green salad vegetables, with the exception of the continental variety, which showed the highest levels.

Low phosphorus and low nitrogen often cause anthocyanin accumulation in plants. Under phosphorus and nitrogen limitation, plants adapt to environmental stress by modulating nutrient partitioning and metabolic pathways, including increased anthocyanin synthesis.

Various factors, including genotype, cultivar, growth stage, and other cultivation conditions, influence the total phenolic content and antioxidant potential of lettuce [3,10,75]. The phenotypic properties of lettuce show significant differences in antioxidant capacity and phenolic content. For example, the mean TPC and antioxidant potential of light and very light red-pigmented samples were significantly lower than those of medium and dark or very dark samples. Broadly elliptical and round-leaf-shaped samples were found to be superior to medium elliptical and broadly ovate leaf shapes. The mean TPC and ABTS values were also significantly higher in highly wavy leaf samples compared to those with medium and slightly wavy leaf margins. The high levels of antioxidants and total polyphenols in the lettuce samples significantly enhance their nutritional value.

Three anthocyanins (cyanidin 3-O-glucoside, cyanidin 3-O-(3-O-malonyl)glucoside, and cyanidin 3-O-(6-O-malonyl)glucoside), four hydroxycinnamoyl derivatives (3-O-caffeoylquinic acid, 5-O-caffeoylquinic acid, 3,5-diO-caffeoylquinic acid, and 2,3-di-O-caffeoyltartaric acid), two flavonols (quercetin 3-O-glucuronide and quercetin 3-O-(6-O-malonyl)glucoside), and a flavone (luteolin 7-O-glucuronide) were identified and quantified in red-pigmented lettuce samples in [76]. In addition, TPC and antioxidant activity were evaluated. A huge diversity of biochemical traits was observed among the lettuce samples. Cyanidin 3-O-(6-O-malonyl) glucoside, 2,3-di-O-caffeoyltartaric acid, and quercetin 3-O-(6-O-malonyl) glucoside were the most dominant in the red-pigmented lettuce samples among the metabolite groups.

The genetic potential of health-beneficial metabolites in the baby leaves of 23 diverse lettuce cultivars was explored in [3]. The study revealed significant variations in metabolite composition and content among lettuce cultivars, primarily influenced by leaf color. Red-leaf cultivars were notably rich in carotenoids (especially all-E-lutein, all-E-violaxanthin, and all-E-lactucaxanthin), polyunsaturated fatty acids (mainly α-linolenic and linoleic acids), total phenolic content, and antioxidant potential. Cyanidin and other phenolic compounds emerged as the strongest radical scavengers based on PCA analysis. Additionally, total folate content ranged from 6.51 to 9.73 μg/g (DW), depending on the cultivar. These findings highlight red-leaf lettuce as a nutrient-rich food with a unique phytochemical profile.

Lettuce quality is one of the hot topics in modern science. The aim of the study by Medina-Lozano et al. [77] was to accurately determine the amount of vitamin C and anthocyanins in lettuce wild relatives, as well as in commercial and traditional varieties used in cultivation. A total of 30 *Lactuca* accessions were studied: 10 commercial lettuce varieties (4 green and 6 red), 13 traditional lettuce varieties (9 green and 4 semi-red), and 7 wild relatives. Among the lettuce varieties, 6 of the 12 types defined by UPOV (2019) were represented: Buttherhead, Batavia, Frisee d’Amerique, Lollo, Cos, and Gem. One of the wild species closely related to *L. sativa* is included in the primary gene pool (*L. dregeana* DC.), while the others are more distant, forming part of the secondary or tertiary gene pool (PGR-lettuce, available also online at https://www.pgrportal.nl/en/lettuce-genetic-resources-portal.htm, accessed on 14 January 2025). It was found that the wild relatives of lettuce and the commercial cultivars were richer in vitamin C and anthocyanins, respectively, with the wild species and the traditional cultivars containing more total ascorbic acid (TAA) than the commercial cultivars (21% and 8%, respectively). Conversely, the commercial cultivars and the wild relatives were the poorest groups in terms of vitamin C and anthocyanins, respectively, with the traditional cultivars occupying an intermediate position in both cases. Green lettuce had a significantly higher TAA content than red lettuce (18%). The leaves of the two wild species also contained significantly more vitamin C than lettuce stems. Cyanidin 3-O-(6′-O-malonylglucoside) was the most abundant anthocyanin (97%), present in most samples.

Some of the traditional lettuce varieties studied in this study were found to be promising because they are rich in vitamin C and in some cases are capable of biosynthesizing anthocyanins, the content of which can be increased by breeding, which is a relevant and “useful” direction from a human nutrition perspective. Some consumable (but not particularly nutritious) leafy vegetables and their wild relatives have potential culinary applications. This may be useful for both consumers and plant breeders interested in improving the quality and content of lettuce in terms of phytochemicals that promote health and reduce the risk of certain diseases in the population.

Thus, several factors may contribute to the large variations in the phytochemical composition and color of lettuce, including the genotype of the cultivar. The intensity of red color, leaf shape, plant growth type, incision density, and leaf margin waviness have been found to influence the concentration of metabolites. Lettuce plants with a high intensity of red color in their leaves, round leaf shape, high leaf waviness, and very dense leaf incisions were found to accumulate the highest concentration of flavonoids and hydroxycinnamic acids. Overall, a number of studies have shown that red lettuce may be one of the main dietary sources of antioxidants, such as caffeic acid and its derivatives.

## 6. Genetic and Epigenetic Control of Anthocyanin in Lettuce

The molecular genetic basis of anthocyanin biosynthesis has been studied quite thoroughly, which has been greatly facilitated by mutants of various plant species with altered coloration. Anthocyanin biosynthesis, and consequently coloration, is affected by mutations in three types of genes: the first are genes that code for enzymes involved in the chain of biochemical transformations (structural genes); the second are genes that determine the transcription of structural genes at the right time in the right place (regulatory genes); and the third are genes of transporters that carry anthocyanins into vacuoles. It is known that anthocyanins are oxidized in the cytoplasm and form bronze-colored aggregates that are toxic to plant cells [55].

As shown in [76], when grown under optimal conditions, wild lettuce typically develops green leaves with an intermittent accumulation of anthocyanins near the leaf margin. Stress, such as drought, promotes anthocyanin accumulation, resulting in a noticeable red coloration of wild lettuce leaves. However, some lettuce cultivars have lost this stress response due to loss-of-function mutations in the gene encoding bHLH. On the other hand, some lettuce cultivars develop red leaves when grown under optimal conditions.

Recent studies have shown that light signals regulate anthocyanins through CONSTITUTIVE PHOTOMORPHOGENIC1 (COP1), which is a negative regulator and mediates the degradation of positive regulators of anthocyanins such as PAP1 and PAP2 in *Arabidopsis* or MdMYB1 in apple [78,79]. Most experiments conducted to study light signal transmission are analyzed by excluding light. However, it was shown in [51,80] that the COP1/SUPPRESSOR OF PHYA (SPA) complex may not be completely inactivated at low light intensity, suggesting the existence of some other light-intensity-induced mechanism in anthocyanin accumulation.

Wada et al. [37] showed that the quantitative analysis of phenolic compounds revealed a genotype-dependent accumulation of anthocyanin in a given artificial environment. The integrated analysis of anthocyanin levels and RNA-seq data collected from nine cultivars with different genotype combinations revealed that, in addition to anthocyanin biosynthetic genes, the transcript levels of a group of genes including *RLL1* (*bHLH*) and *RLL2* (*MYB*) were highly correlated with anthocyanin levels. This result indicated that the genetic network regulating the activity of the *MYB-bHLH-WD40* (MBW) complex plays a crucial role in anthocyanin accumulation in lettuce under artificial light.

Leaf color is an important factor influencing consumer acceptance of red-leaf lettuce, and light significantly influences leaf color [55]. It was shown in [81,82] that red-leaf lettuce had a greener leaf color when grown under controlled conditions at a light intensity of 40 mmol m^−2^ s^−1^, whereas increasing the light intensity to 100 mmol m^−2^ s^−1^ resulted in lettuce with a redder leaf color. This red color is known to be due to increased anthocyanin accumulation [83]. However, the molecular mechanisms governing light-induced anthocyanins in red-leaf lettuce are still not fully understood.

Anthocyanin biosynthesis has been studied quite extensively in *Arabidopsis*, and more than 29 anthocyanin molecules have been identified in *Arabidopsis* [82], regulated by strong light alone or in combination with low temperature exposure. However, the regulation of phytochemical biosynthesis and its accumulation, as well as the underlying molecular mechanisms of light-induced phytochemical biosynthesis, are poorly understood in lettuce.

The constitutive up-regulation of anthocyanin biosynthesis is mediated by a gain-of-function mutation in the gene encoding the MYB transcription factor and loss-of-function mutations in two negative regulators [76,84]. Thus, green-leaf and red-leaf lettuce cultivars have been selected both during domestication and in modern breeding programs, demonstrating typical artificial disruptive selection, i.e., selection favoring extreme values over intermediate values [6,33]. Varieties lacking anthocyanins may have a reduced ability to withstand biotic and abiotic stress. On the other hand, varieties with high concentrations of anthocyanins in epidermal cells will block light penetration and, therefore, reduce photosynthesis and plant growth. However, selection based on green and/or red vegetable leaves is carried out by humans, and is artificial.

Zhang et al. [85] found that different lettuce species exhibit enormous morphological variations. However, the molecular basis of domestication and divergence among different garden lettuce species remains unknown. Zhang et al. [85] used 240 lettuce samples collected from major garden species and wild relatives for RNA sequencing, generating 1.1 million single-nucleotide polymorphisms (SNPs). The demographic modeling suggested that there was a single domestication event for lettuce. Several genome regions demonstrated putative selective sweeps that occurred during domestication and divergence, respectively. Genome-wide association studies (GWAS) identified 5311 expression quantitative trait loci (eQTL) regulating the expression of 4105 genes, including 9 eQTL regulating genes related to flavonoid biosynthesis. The study showed that six potential loci responsible for anthocyanin variation could be identified in lettuce leaves.

Using gene annotation and phylogenetic analysis, putative structural genes involved in anthocyanin synthesis and transport were identified, as presented in Table 2 (adapted from Table 1 in [51]). Their NCBI GenBank accession numbers are MF579543–MF579560. The authors of the study identified nine key genes involved in anthocyanin biosynthesis: seven anthocyanin structural genes, including *CHS*, *CHI*, *F3H*, *F3′H*, *DFR*, *ANS*, and *3GT*, and two anthocyanin transport genes, *GST* and *MATE*. In addition, six anthocyanin regulatory genes were identified: five *MYBs* and one *bHLH* gene.

Anthocyanin biosynthesis in plants is mainly controlled by structural anthocyanin biosynthesis genes, which are divided into two groups: early biosynthetic genes (EBGs, such as *CHS*, *CHI*, and *F30H*) and late biosynthetic genes (LBGs, such as DFR *LDOX*, *UF 3 GT*, *UGT 75 C 1*, and *3 AT 1*) [51]. EBGs encode key enzymes for the synthesis of precursors common to flavonoids or other phenolics, while LBGs encode enzymes specific to anthocyanins [19]. Thus, it can be concluded that anthocyanin biosynthesis in plants is mainly regulated by the MBW complex [51,86]. The *HY5* gene was discovered, which can respond to light signals and regulate the structural genes of anthocyanins. These genes were significantly overexpressed (log2FC = 2.7–9.0) under high irradiance and were confirmed by real-time quantitative polymerase chain reaction (RT-qPCR) [51].

Although the biosynthesis of anthocyanins is regulated mainly by the MBW complex, it is also greatly influenced by various environmental factors (e.g., light, salinity, drought, cold) and phytohormones (e.g., jasmonate, abscisic acid, and auxin). Advances in understanding these regulatory networks highlight anthocyanins’ potential applications in agriculture, horticulture, and the food industry [86].

Damerum at el. [87] studied lettuce recombinant inbred lines generated from a cross between wild and cultivated lettuce (*L. serriola* and *L. sativa*, respectively). Antioxidant (AO) potential and important phytonutrients, including carotenoids, chlorophyll, and phenolic compounds, were analyzed. When grown in two environments, 96 quantitative trait loci (QTL) were identified for these nutritional traits: 4 for AO potential, 2 for carotenoid content, 3 for total chlorophyll content and 87 for individual phenolic compounds. Most often, the *L. serriola* alleles conferred an increase in total AOs and metabolites. Candidate genes underlying these QTL were identified by BLASTn searches; in several cases, they had functions suggesting involvement in phytonutrient biosynthetic pathways. The analysis of QTL on linkage group 3, which accounted for >30% of the variation in AO potential, revealed several candidate genes encoding multiple MYB transcription factors, which regulate flavonoid biosynthesis and flavanone 3-hydroxylase. The latter enzyme is involved in the biosynthesis of the flavonoids quercetin and kaempferol, which are known to have powerful AO activity. The follow-up RT-qPCR of these candidates revealed that 5 out of 10 genes investigated were significantly differentially expressed between the wild and cultivated parents, providing further evidence of their potential involvement in determining contrasting phenotypes.

Studies of the genetic variations that contribute to the diversity of leaf color in lettuce cultivars and the expression analysis of genes involved in anthocyanin biosynthesis highlight the complex regulatory network governing pigment production. Meanwhile, understanding the genetic control of anthocyanin biosynthesis in lettuce provides valuable insights for breeding programs aimed at developing cultivars with desirable pigmentation and enhanced health benefits.

## 7. Practical Recommendations for the Selection and Cultivation of Red-Leafed Lettuces

During lettuce growth, whether grown outdoors, indoors, or in vertical farms, each leaf of the plant has different levels of exposure to environmental conditions (light, humidity, nutrient uptake, and temperature), and the inner leaves are younger than the outer leaves. These factors may influence the distribution of phytochemicals and antioxidant capacity in the above-ground portion of the lettuce. Knowledge of the bioactive content and antioxidant capacity profile of lettuce plants may be of interest to both consumers and the food industry in order to select more suitable leaves or cultivars for salads or other ready-to-eat mixed vegetable dishes with high nutritional value.

The information presented in this review summarizes the current research on the influence of various factors on certain plant species and discusses the use of LED lighting in horticulture, crop storage, and disease control; it is in these areas that it will be especially important to consider the fact that the value, quality, and weight of grown products are the result of the genotype and abiotic factors, which are either created and controlled by the agronomist or not controlled in the open ground, but used to manage the quality of grown products. LED lamp control allows researchers to combine significant reductions in energy losses and controlled plant growth, achieving the improvement of two main parameters in one action.

The information presented in this review may be potentially useful for advertisers and healthcare providers to promote and develop a food culture of consuming red leafy plants, particularly lettuce, by increasing the levels of pigments with nutritional value (e.g., beta-carotene and anthocyanins) in foods grown on vertical farms or in soil.

## 8. Conclusions

This review presents a comprehensive analysis of modern approaches to studying the uniqueness and value of red-leaf lettuces, highlighting their practical application and potential for advancing cultivation technologies. Red-leaf lettuces stand out due to their exceptional aesthetic and functional properties, offering a rich source of bioactive phytometabolites such as anthocyanins, flavonoids, and carotenoids. These compounds not only contribute to the vibrant red coloration, but also play a significant role in improving human health by boosting immunity and reducing the risks of oxidative-stress-related diseases. The ability to manipulate plant traits such as color, taste, size, and productivity by leveraging cultivation technologies underscores the versatility and adaptability of red-leaf lettuce as a crop. The summarized findings are presented in Table 3, which outlines that both genetic and epigenetic or environmental factors influence photomorphogenesis, which regulates leaf coloration and phytochemical content.

The cultivation of red-leaf lettuces in controlled environments, such as vertical farms, offers significant advantages for meeting consumer and market demands. Technologies like LED lighting enable precise control over phenotypic traits, allowing growers to optimize color intensity, antioxidant content, and nutritional value. Low root-zone temperatures and carefully tailored light spectra have been shown to enhance the accumulation of anthocyanins and other valuable compounds, while maintaining acceptable levels of plant productivity. These insights are crucial for developing high-value products tailored to the preferences of consumers and the requirements of industrial partners, such as LLC “Zelen”, who aim to commercialize red-leaf lettuce production in vertical farming systems.

Furthermore, this review emphasizes the importance of promoting a dietary culture that includes green crops, particularly red-leaf lettuces, due to their health benefits and rich phytochemical profiles. Consumers, breeders, and nutraceutical companies are encouraged to focus on key phenotypic traits—such as color intensity, leaf shape, and phenophase transitions—as well as abiotic conditions like temperature, pH, and light spectrum. These factors provide opportunities to enhance the genotype’s potential and produce crops with superior nutrient content and functional qualities.

Prospective studies should integrate these findings into practical agronomy to address both consumer preferences and market demands. An example of such studies is the research that is planned to be conducted in the City Farming Laboratory of the Gastronomy Institute at Siberian Federal University (Krasnoyarsk, Russia; https://www.sfu-kras.ru/en/news/29418, assessed on 16 January 2025). This initiative aims to establish sustainable production technologies for red-leaf lettuces in vertical farming systems, creating innovative solutions for the restaurant industry and promoting functional foods in everyday diets. This approach combines cutting-edge cultivation practices with a deeper understanding of genetic, epigenetic, and environmental factors, delivering high-quality products for modern agriculture and gastronomy.

## Figures and Tables

**Table 2:** Differentially expressed genes related to anthocyanin.

Gene Name	Low-Light FPKM	High-Light FPKM	Log 2 Fold Change (Hiht/Low Light)	FDR	Up/Down
*LsCHS*					Up
Unigene12000_All	1.68	858.26	9.0	0	
CL4608.Contig2_All	11.36	97.28	3.1	5.33 × 10^−259^	
*LsCHI*					Up
Unigene10166_All	10.6	68.72	2.70	1.43 × 10^−96^	
*LsF3H*					Up
Unigene8465_All	14.21	408.44	4.85	0	
*LsF’3H*					Up
CL524.Contig1_All	9.08	193.8	4.42	0	
*LsDFR*					Up
Unigene2105_All	2.3	473	7.68	0	
*LsANS*					Up
CL1994.Contig1_All	4.25	269.69	5.99	0	
*Ls3GT*					Up
CL4808.Contig1_All	5.84	158.28	4.76	0	
CL4808.Contig2_All	3.31	84.6	4.66	0	
*LsGST*					Up
Unigene10814_All	1.84	245.41	7.06	0	
*LsMATE*					Up
Unigene12020_All	8.42	18.14	1.11	9.25 × 10^−15^	
*LsMYB*					
Unigene12430_All	2.21	8.30	1.91	4.36 × 10^−10^	
Unigene12294_All	3.14	37.14	3.56	5.02 × 10^−85^	Up
Unigene23058_All	0.11	7.25	6.04	5.68 × 10^−17^	
Unigene24751_All	0.56	22.95	5.36	7.05 × 10^−86^	
CL6440.Contig1_All	0.72	4.60	2.66	9.14 × 10^−11^	
*LsbHLH*					Up
Unigene13011_All	3.64	21.95	2.59	3.37 × 10^−84^	
*LsHY5*					Up
Unigene19629_All	3.18	10.42	1.71	3.15 × 10^−5^	

**Table 3:** The main genetic and epigenetic or environmental factors influencing photomorphogenesis that regulate leaf coloration and phytochemical content.

Factor	Description of the Factor	Effect on Coloring	Mechanism of Action	Reference
**Genetic factors**				
Mutations in *RLL1*, *RLL2*, *RLL3*, and *RLL4* genes that encode *bHLH*, *R2R3-MYB*, *R2-MYB*, and WD-40 transcription factors, respectively	Mutations that result in gain or loss of gene function	Strengthening or weakening of red color	Changes in the transcriptional activity of genes, controlling anthocyanin biosynthesis	[76]
Gene changes and losses during domestication	Loss of function of wild-type genes during cultivation	Loss of red color, appearance of green varieties	Decreased or stopped synthesis of anthocyanins	[29]
Genetic diversity of varieties	Differences between varieties and genotypes	Variation in the content of anthocyanins and phenolic compounds	Genetic predisposition to accumulation of phytochemicals	[74]
**Epigenetic or environmental factors**				
Light spectrum	Exposure to different light spectra (blue, red, UV-A)	Strengthening or weakening of red color	Regulation of anthocyanin biosynthesis gene expression through photomorphogenesis	[30]
Root zone temperature	Changing the temperature of the nutrient solution (low or high)	Enhanced anthocyanin accumulation at low temperature; variable effect at high temperature	Induction of stress reactions, increase in antioxidant synthesis	[56]
pH of the environment	Changing the acidity of the nutrient medium	Effect on antioxidant activity and possible color change	Modification of element bioavailability and enzyme activity	[18]
Position of the leaf on the plant (ecological)	Inner, middle, or outer leaves	Change in color intensity and pigment content	Differences in light exposure and tissue age	[30]

## Data Availability

Data are contained within the article.
